# “That’s Me I am the Farmer of the Land”: Exploring Identities, Masculinities, and Health Among Male Farmers’ in Ireland

**DOI:** 10.1177/15579883211035241

**Published:** 2021-08-20

**Authors:** Conor Hammersley, Noel Richardson, David Meredith, Paula Carroll, John McNamara

**Affiliations:** 1 Department of Science and Health, National Centre for Men’s Health (NCMH), Institute of Technology, Carlow, Ireland; 2 Teagasc, Ireland’s agriculture and food research and development authority, Ashtown Research Centre, Ashtown, Dublin, Ireland; 3 Centre for Health Behaviour Research, Waterford Institute of Technology, Waterford, Ireland

**Keywords:** farmers’ health, farming masculinity, farming governance, farmer stress

## Abstract

Compared to other occupational groups, farmers in Ireland experience a disproportionate burden of health problems, which impact farmers’ livelihoods and farming sustainability. Internationally, farmers’ poor health outcomes are associated with intersecting economic, environmental, socio-cultural, and occupation-specific factors linked to changes in agricultural governance. This qualitative study explored the challenges and stressors facing farmers in Ireland and how changes in farming governance have impacted farmers’ identities, masculinities and health. Eleven focus groups (*n* = 26 female, *n* = 35 male, age-range 20s–70s) were conducted with both male farmers (*n* = 3 focus groups; *n* = 13) and key informants (*n* = 8 focus groups; *n* = 48, 22 male, 26 female). Utilizing Thematic Content Analysis, transcripts were coded independently by the first and second author using open and comparative coding techniques, with emerging themes grouped into primary and subthemes. Theme memos and conceptual maps tracked evolving relationships between themes. The analysis identified three broad themes. “Wrestling with challenges to autonomy and control within farming” examines the impact of tighter regulatory frameworks associated with changes to farming governance and unpacks other challenges associated with scale and succession. “Farming masculinities and health” explores how farming masculinities were closely aligned with farming practices and health practices and were framed relationally. “Isolation and the demise of rural communities” considers the impact of reduced social interaction on loneliness among farmers, particularly among more “at risk” single and older farmers. Findings provide unique insights into contemporary challenges and stressors facing farmers and have important implications for informing the design and roll-out of a national farmers’ health training program.

Internationally, farming has long been regarded as a stressful occupation prompting widespread concern for farmers’ well-being (Kennedy et al., 2020; Lunner Kolstrup et al., 2013). The diverse and complex range of stressors associated with farming has resulted in farming becoming synonymous with more adverse health outcomes relative to other occupations (Health and Safety Authority [HSA], 2015). The focus of this study is on male farmers’ health. Notwithstanding the unique challenges that impact on female farmers’ health (Shortall et al., 2017), the rationale for focusing on male farmers’ health is grounded in (i) particularly adverse health outcomes for male farmers related to cardiovascular disease (Smyth et al., 2013; van Doorn et al., 2017), cancers (Smyth et al., 2013) and mental health (O’Donnell & Richardson, 2018; Storey et al., 2014); (ii) calls at a national men’s health policy level for more targeted and gender-sensitive approaches to male farmers’ health (Richardson & Carroll, 2009); and (iii) efforts, as part of a wider study, to inform the design of a farmers’ health training program under the auspices of ENGAGE—a national men’s health training program (Carroll et al., 2020; Lefkowich et al., 2018; Osborne et al., 2018). Farmers’ poor health outcomes have a detrimental impact on farmers’ lives, resulting in higher risk of farming injury, disability, premature death, and loss of farm profitability (Bloom et al., 2011). Indeed, in the context of declining mortality rates more generally in Ireland over recent decades, mortality rates among Irish farmers have decreased the least of all socio-economic status (SES) groups (Layte et al., 2015). This, as Smyth et al. (2013) argue, is a direct reflection of the decline in farm incomes during the same period and warrants a more targeted policy response and prioritization of farmers and agricultural workers as a high-risk group. International research has linked these adverse health outcomes to a variety of factors, such as solitary work, high workload and time pressure, machinery breakdown, unfavorable weather conditions, and financial aspects, for example, irregular and uncertain income, and financial debt (Letnes et al., 2016; Lunner Kolstrup et al., 2013; Walker, 2012). Additionally, many social difficulties associated with farming have been noted, such as balancing work and family and working with multigenerational family members (Cassidy, 2017; Roy et al., 2017). Emerging literature (Burns, 2021; Burton et al., 2008; Forney, 2016; Sutherland & Darnhofer, 2012) draws attention to changes in agriculture governance as a critical source of stress for farmers. Specifically, the shift from a “productivist” focus on intensification—associated with negative environmental externalities within large scale farming operations (predominantly cattle feedlots, large-scale poultry and grain operations) to more “agri-environmental” farm practices (e.g., price supports for biodiversity development, upkeep of rivers, reducing greenhouse gases, and animal/plant welfare) has had significant implications for farmers’ work practices and livelihoods.

## Farming Identities and Farming Masculinities

This changing nature of farming at a policy level has received mixed responses from the farming community—with many farmers demonstrating resistance to such change (Forney, 2016). Many of the challenges arising from changes to farming governance are compounded by aspects of farmer identity and more dominant or “hegemonic” constructions of farming masculinity. Connell (1995) theorized that hegemonic masculinity refers to the culturally exalted position afforded at any given time to one form of masculinity over others, and reflects men’s domination of women and a complex system of inter-male dominance. The pursuit of hegemonic masculinity is therefore synonymous with the pursuit of power and is defined against a range of subordinated and often stigmatized masculinities. Connell also stressed that hegemonic masculinity is not a fixed character type but rather represents “configurations of practice” that are contextual and fluid. In other words, men construct masculinities in accordance with their position within social structures, and their access to power and resources that such positions afford them. Indeed, as the circumstances or conditions that uphold a particular masculinity are challenged, hegemonic masculinity needs to be reconfigured in order to sustain its dominant position. Subsequent seminal work (Courtenay, 2000, 2011; Mahalik et al., 2003; O’Brien et al., 2005) highlighted how constructions of “hegemonic masculinity” were linked to health practices and predicated on, among other behaviors, the suppression of emotions and help-seeking. The contention in these studies was that the pursuit of “hegemonic” masculinity was typically at the expense of health or engaging in health-enhancing behaviors—with Courtenay, (2011) stating that men may deliberately engage in health-damaging behaviors to actively perform masculinities and reinforce their masculine identity.

An exploration of farming masculinities offers important insights into how farmers relate to health and how the environment in which they live and work shapes this relationship. Research from Ní Laoire (2005) found that the pursuit of more dominant or “hegemonic” farming masculinity within an Irish context revolved around the farmer as a hard worker, battling against environmental/economic obstacles, exerting “his” authority over the natural landscape, and prioritizing farm work over self-care. Similarly, in a Canadian context, Roy et al. (2017) found that farmers often sacrificed rest in favor of a “relentless worker” model to uphold their masculine image. Roy and Hočevar (2019) note how farmers are continually negotiating health practices in relation to hegemonic masculinity; and that “doing health” is a form of “doing gender.” Indeed, the act of opposing health-enhancing behaviors or help-seeking behavior could be seen as an active demonstration of manliness for some farmers and a rejection of the more feminine connotations of weakness and stigmatization associated with health (Ní Laoire, 2005; Roy et al., 2014, 2017). In this sense, the cultural influences that shape farmers’ beliefs, attitudes, and practices are synonymous with the pursuit of a hegemonic form of farming masculinity that can be demonstrated in the avoidance of behaviors that show concern for health or that require emotional expression or help-seeking on the basis that such practices might position farmers as weak or vulnerable (Cleary, 2012; Ní Laoire, 2005; Roy et al., 2017; Verdonk et al., 2010). This does not imply that masculinity in a farming context is a fixed or consistent concept (Cush & Macken-Walsh, 2018; Roy et al., 2017), but rather that masculinities of various kinds can attain relative solidity in certain socio-cultural contexts and can be more fluid in other contexts (Cleary, 2005, 2012; Cush & Macken-Walsh, 2018).

Burton’s concept of “the good farmer”—rooted in symbolic demonstrations of self-reliance, combatting adversity, and suppressing emotions—is intertwined with farming identities, farming practices, and farmers’ sense of self-worth (Burton, 2004). The term seeks to capture the sets of behaviors and attitudes adopted, resisted or rejected by farmers in the acquisition of objectified cultural capital through symbolic means, visible in conventional farming cultures—primarily through symbols of production—for example, large grain operations (Burton et al., 2021; Rogers, 1983), modern machinery (Ní Laoire, 2005) or the presence of quality livestock or crops (Sutherland & Burton, 2011). The acquisition and maintenance of such symbols are synonymous with status within one’s community (Sutherland & Burton, 2011). Ward (1993) conceptualizes this as a product of the intensification of production, underpinned by increased mechanization and technological use, leading to an increased income for farm households. Indeed, Brandth (1994) observes that the increased mechanization that accompanied the rise in productivist agriculture, was also defined by a new masculinization of farming—in that, machinery became symbolic of power, control and technical skill for the farmer (Ní Laoire, 2005). This intensification of farm practices became embedded in farming culture—in that, acquiring cultural capital to achieve “good farmer” status became entrenched in productivist agriculture methods (Burton et al., 2008). However, the European Union (EU) agricultural policy reforms in the early 1990s—prompted, in particular, by environmental concerns at a wider societal level (Department of Agriculture, 2000) —contested this large scale agricultural production model in favor of a range of agri-environmental policy measures. These measures lowered the price supports for grain, beef and dairy production to curb excessive EU agricultural production, which was deemed to be negatively affecting the environment and animal welfare. This marked the phasing out of direct payments for production—a development referred to as “decoupling” —in favor of a single farm payment (SFP) system (Hennessy & Kinsella, 2013). The SFP was subject to farmers’ adherence to good agri-environmental practices in relation to biodiversity development, upkeep of rivers, animal and plant welfare, and critically included regulation and inspection from authorities (Hennessy & Kinsella, 2013).

While some farmers willingly accepted these changes, others resisted, and tensions emerged between the traditional farmer and contemporary society (Burton et al., 2021). The new role expected of farmers—to be custodians of the natural landscape and environment—was a significant departure from the historically symbolic expressive methods that attained social status and recognition by standards of “the good farmer” (Burton & Wilson, 2006; Sutherland & Burton, 2011). Resistance to these new agri-environmental measures associated with “the good farmer” has strong links to the construction of masculinity in farming (Burton et al., 2008; Peter et al., 2009). Tensions persist therefore between traditional and contemporary constructions of farming masculinity against a backdrop of significant shifts in agricultural policy. In particular, there is an apparent contradiction between farmers’ self-representations as autonomous and independent on the one hand and their clear obligations to be compliant with and subservient to regulations on the other (Forney, 2016). As farmers struggle to navigate an increasingly complex governance environment and a precarious natural environment, many are left questioning the very essence of who they are and whether their concerns are aligned with the goals of wider society (Cassidy, 2014).

Traditionally, there has been a strong gendered dimension to agriculture and to the division of farming roles and responsibilities within farming families. Examples include the patrilineal line of inheritance and gender inequalities in ownership of farm assets, the invisibility of women’s farm work, the continuing male domination of farming organizations and the farming media, and the assumption that women assume the caring and nurturing responsibilities in farming households (Byrne et al., 2013; Shortall et al., 2017). Emerging literature suggests that despite increasing female representation in the Irish labor market and changes to the position of females within the family farm—with more females taking up off farm employment (CSO, 2016), such changes have been slow to cascade into a reconfiguration of farming roles and responsibilities. For example, Shortall et al. (2017) reported that female partners in a farming household are acutely aware of the challenges posed to male farmers’ identity and health, and will actively assume a more subordinate role in order to elevate the identity of the “strong male provider,” and to support his self-worth. Hence, male farmers’ pursuit of a particular masculine ideal as “the provider” can be reinforced by female partners. It has been argued that this emphasis on autonomy, stoicism, and self-reliance associated with the construction of hegemonic masculinity fuels male farmers’ negative attitudes toward emotional expression and help-seeking (Fraser et al., 2005; Roy et al., 2014; Roy & Hočevar, 2019). It is crucially important therefore to frame farming masculinities within a gender relations context and to consider the perspectives of wives/partners of farmers in co-constructing the relationship between farming masculinities and health.

## Changes in Farming Governance and Impact on Rural Communities

The long-running process of agricultural restructuring has cascaded into a range of collateral impacts on farmers and the wider fabric of rural communities, including challenges related to farm succession, migration from rural communities, increased mechanization and a rise in isolation and loneliness. A UK study, for example, noted how socio-economic struggles had prompted a cultural shift in farming with parents encouraging their children to pursue education over farm succession (Price & Evans, 2009). Similarly, in Ireland, this has signified a shift away from traditional farm succession and outward migration of young males to urban areas (Cassidy, 2014). This decline in farm successors has increased the solitary nature of farming, particularly for older farmers who feel duty-bound to stay farming the land despite their diminishing capabilities to do so (Amshoff & Reed, 2005). Additionally, increased mechanization has reduced the need for physical manual labor—hence, farming has not only become less physically active but more solitary and isolating (Ní Laoire, 2005; Roy et al., 2013). Chronic social isolation has been noted as a high-risk factor for loneliness and poor mental and physical health (Cacioppo, 2011; Cacioppo & Hawkley, 2009; NASEM, 2020). The consequences and cost of loneliness to the individual can be far-reaching and include disruption of social interactions and routines, reduced meaningful activity and reduced social and emotional support (Cacioppo et al., 2009). Hence, loneliness and its consequent impact on poor mental health in farming have been attributed to a complex range of interconnected cultural, social, and psychological risk factors (Gallagher & Sheehy, 1994; Lefkowich et al., 2017; Shortall, 2014). Different manifestations of loneliness have been attributed to deficits in relationships at intimate partner, close friendships and wider community levels (Murthy, 2020).

In addition to understanding what stressors are most strongly associated with changes to farming governance, it is also important to understand how stress manifests and is mediated by other factors. The stress process theory (Pearlin et al., 1981), which focuses attention on the sources, mediators, and manifestations of stress, helps explain how the challenges farmers face can affect their psychological well-being. Sources of stress can be either acute (e.g., economic market changes (Sunderman & Johannes, 2019)) or chronic (e.g., loss of autonomy associated with agricultural policy reforms (Forney, 2016)). Mediators of stress include, for example, social support and coping (Minnotte & Yucel, 2018; Sunderman & Johannes, 2019). In more remote rural areas, where access to social support can be limited, manifestations of stress include increased rates of depression and suicide, associated with reduced coping mechanisms (Kennedy et al., 2020; Mcintosh et al., 2016).

## The Present Study

In summary therefore, while farmers’ well-being has always been exposed to a range of occupational stressors, the nature and implications of these stressors are evolving. Compounding this, emerging literature suggests that farmers’ sense of identity and masculinity, in particular, are being increasingly challenged by the evolution of agricultural policy. Critically, when the ideals and practices upon which farmers have built their reputation are no longer appreciated by wider society, this may have negative consequences for farmers’ mental health. This is particularly problematic given deeply entrenched masculine norms among the farming community, which militate against farmers asking for help or accessing support. Notably, within an Irish context, there is a gap in the literature on the challenges and stressors currently facing farmers within the wider context of rural communities, the factors underpinning these, how they impact on Irish farmers’ health, the socio-cultural barriers that reduce farmers’ adaptive capacity to deal with these stressors, and ultimately how these issues can be addressed. This study sought to address this gap by exploring “male farmers” and other “key informants” experiences and perspectives in relation to these issues within an Irish context. In doing so, we sought to address three particular research questions; (i) how have changes in farming governance impacted on farmers in terms of contemporary challenges and stressors; (ii) what are the ripple effects of these challenges on farmers’ identities, masculinities and health and is it time to contest the notion of the “the good farmer”; and (iii) how have changes to farming governance impacted more broadly on rural communities. This study is part of a wider study commissioned to inform the design of a bespoke farmers’ health training program.

## Methodology

This study adopted a community-based participatory research (CBPR) approach (Israel et al., 2008). This involved collective and systematic inquiry in which researchers, statutory organizations, and community stakeholders engaged as equal partners in all steps of the research process with the goal of improving practice in relation to farmers’ health (Baum et al., 2006; Israel et al., 2008; Jull et al., 2017). The study was overseen by a multi-stakeholder advisory group comprising representatives from two government departments, the national health service, four non-governmental organizations (NGOs), and academics representing three third level institutes (*n* = 14, see Table 1). This group offered guidance and advice on the research process and design and assisted with recruitment of research participants. Incidentally, four members of the advisory group hail from farming households, with three working as part-time farmers.

**Table 1.** Multi-Stakeholder Advisory Group.

		*N*
Governmental departments	The Health Service Executive (HSE)	1
	The Department of Agriculture, Food and the Marine (part-time farmer *n* = 1)	2
	Teagasc—Ireland’s agricultural and food development authority (part-time farmer *n* = 1)	2
Non-Governmental Organizations (NGOs)	The Men’s Development Network	2
	The Irish Heart Foundation	1
	Mental Health Ireland	1
	“Fit for farmers” initiative	1
Academia	Institute of Technology Carlow (farming background *n* = 1)	2
	Waterford Institute of Technology (part-time farmer *n* = 1)	1
	Nuffield scholar (part-time farmer *n* = 1)	1
**Total**		**14**

The study was approved by the Institute of Technology Carlow’s Ethics Committee (Ethical Application Number 252). Eleven focus groups (45–60 min; *n* = 29 female, *n* = 35 male, *n* = 63 total) were conducted from Nov 2019–Mar 2020 with male farmers (*n* = 3 focus groups, *n* = 13 participants) and other key informants (*n* = 8 focus groups, *n* = 48 participants; 22 male, 26 female) (Table 2). Participants were recruited via purposive sampling (Silverman, 2000). The three male farmer focus groups constituted participants from three different farming enterprises—(i) dairy farmers^1^ (*n* = 3), (ii) dry-stock farmers^2^ (*n* = 5), and (iii) tillage/stock farmers^3^ (*n* = 5)). The key informants constituted agricultural advisors from dairy (*n* = 8), dry-stock (*n* = 4) and tillage (*n* = 7) backgrounds, wives/partners (working on farm, *n* = 7; working off farm, *n* = 5) and farming representative organizations (Irish Farmers Association, *n* = 6; Macra na Feirme^4^, *n* = 5). A gatekeeper for each focus group was initially contacted by the lead author or by an advisory group representative. Gatekeepers were identified on the basis of being strategically positioned to provide access to and help convene a particular target group. For example, a regional agricultural advisor helped convene the tillage and dry-stock farmer groups; a member of the advisory group helped convene the dairy farmer group; while different members of the advisory group helped convene the key informant groups. After participants agreed, informally, to take part in the study via their respective gatekeeper, the lead researcher sent an information sheet and consent form to participants via email. All signed consent forms were returned to the lead author prior to each focus group commencing.

**Table 2.** Overview of Focus Group Participants.

**Focus Group**	**Target Group** (Geographical location)	**Participants code** (Age category of participant)	**Participants** ***N***	**Males *N***	**Females** ***N***
1	**Dairy farmers** (Mid-East of Ireland)	**F001** (40–49), **F002** (40–49), **F003** (50–59)	3	3	0
2	**Dry-stock farmers** (West of Ireland)	**F004** (50–59), **F005** (40–49), **F006** (60–69), **F007** (50–59), **F008** (60–69)	5	5	0
3	**Tillage/stock farmers** (South-East of Ireland)	**F009** (30–39), **F010** (40–49), **F011** (30–39), **F012** (30–39), **F013** (50–59)	5	5	0
**Total male farmers**			**13**	**13**	**0**
4	**Dry-Stock advisors** (West of Ireland)	**AA001** (40–59), **AA002** (40–49), **AA003** (50–59), **AA004** (40–49)	4	3	1
5	**Dairy advisors** (South-West of Ireland)	**AA005** (20–29), **AA006** (40–49), **AA007** (20–52), **AA008** (20–29), **AA009** (20–29), **AA010** (40–49), **AA011** (20–29), **AA012** (40–49)	8	4	4
6	**Tillage advisors** (South-East of Ireland)	**AA013** (60–69), **AA014** (30–39), **AA015** (40–49), **AA016** (30–39), **AA017** (20–29), **AA018** (40–49), **AA019** (50–59)	7	7	0
7	**Private advisors** (South of the midlands)	**AC001** (40–49), **AC002** (40–49), **AC003** (40–49), **AC004** (60–69), **AC005** (60–69), **AC006** (50–59)	6	3	3
8	**“Wives/partners” (working on farm)** (South of the midlands)	**F014** (50–59), **F015** (50–59), **F016** (70–79), **F017** (30–39), **F018** (50–59), **F019** (50–59), **F020** (60–69)	7	0	7
9	**“Wives/partners” (working off farm)** (South-East of Ireland)	**FG001** (50–59), **FG002** (50–59), **FG003** (50–59), **FG004** (50–59), **FG005** (60–69)	5	0	5
10	**Irish Farmers Association** (East of Ireland)	**P001** (50–59), **P002** (50–59), **P003** (50–59), **P004** (50–59), **P005** (60–69), **P006** (40–49)	6	0	6
11	**Macra na Feirme group** (South of the midlands)	**MNF001** (20–29), **MNF002** (30–39), **MNF003** (30–39), **MNF004** (20–29), **MNF005** (20–29)	5	5	0
**Total key informants**			**48**	**22**	**26**
**Total participants**			**61**	**35**	**26**

The criteria used for selecting the male farmer groups were based on convening a diverse sample in terms of geographical location, farm enterprise and age-profile. While specific age was not collected due to its sensitive nature (Johnson et al., 2003) and on the advice of the advisory group, age category data from 20 to 80 in 10 year increments was collected. The criteria used for selecting the “key informants” were based on guidance from the advisory group—targeting those who, in a professional and/or a personal capacity, had close relations with male farmers, had first-hand knowledge and experience of the issues that impact on male farmers’ health, and were likely to offer more in-depth insights into the key research questions for the study. Agricultural advisors work closely with farmers offering advice and guidance on agricultural matters, and are generally regarded as close confidants of farmers. The agricultural advisor groups were selected based on diversity in terms of geographical location, farm enterprise, gender and age. The wives/partners (working on farm) and the wives/partners (working off farm) groups were selected based on the traditionally strong gendered dimension to farming roles and responsibilities—including many females acting as custodians of male farmers’ health (Shortall et al., 2017) —and the rationale for framing farming masculinities and health within a gender relations context (Roy et al., 2017; Shortall, 2014). These groups were selected based on diversity in terms of on/off farm work, geographical location and age. The farmer representative organization groups were selected based on their mandate of giving a voice to farmers and representing the interests of different farming sectors. These groups were selected based on diversity in terms of geographical location, gender and age.

### Data Collection

Focus groups were chosen as the preferred data collection tool to explore potentially sensitive topics, whereby individuals with shared identity characteristics could feel empowered and supported in the group setting (Morgan, 2014). A focus group topic guide structured the process for both male farmers and key informants (Table 3). The topic guide was the same for both “male farmers” and “key informants” with the objective being to seek multiple perspectives on the same research questions. This was constructed following an extensive review of the literature and with input from the advisory group. Open-ended questions focused on the impact on farmers of changes to agricultural governance and policy (Burton et al., 2021), broader challenges and stressors facing farmers, links between farmers’ health practices and the construction of farming and rural masculinities (Ní Laoire, 2005; Roy et al., 2017), farmers’ attitudes and approaches to health (Verdonk et al., 2010), and barriers farmers face when engaging with health (Roy et al., 2014). The lead author drafted the focus group topic guide, with cross-reference and reflections from the remaining authorship of this paper until consensus was reached.

**Table 3.** Topic Guide Questions.

Topic	Considerations
Broad description of your job	Responsibilities/tasks
Health (broadly)	Consideration to physical & mental health issues
	Lifestyle issues
	Access to services—barriers to access & awareness of how/where to access services
	Knowledge/awareness of health
	Health beliefs and attitudes to health behaviors like eating habits, exercise, smoking, drinking, and leisure time
Farm enterprise/contemporary challenges and relationship to health	Agricultural governance and policy Factors within and outside of their control Health issues that affect you based on this
Facilitators that support farmers to engage in help-seeking behavior and health?	Access to services Spouse or close confidant Previous health scare
Barriers and facilitators farmers face when engaging in help-seeking behavior and health?	Access to services Time pressures Masculinity Social pressure

The focus group moderator was a young (mid-twenties), heterosexual, Irish white male PhD student from rural Ireland, and who had an interest in farming masculinities and health and had prior experience in qualitative research. The second author, who has over 20 years’ experience in men’s health qualitative research, conducted three workshops with the lead author (moderator), prior to data collection commencing. In accordance with Masadeh (2012, p. 67), these workshops focused on (i) engagement questions: introducing participants to and making them comfortable with the topic of discussion, (ii) exploration questions: probing questions that get to the heart of the discussion and (iii) exit question: closing question that seeks any further comments regarding the topic, and to check if anything was missed. The second author observed the lead author (moderator) facilitate the first two focus groups, and provided detailed feedback after each. At this point the principal investigator was confident in the lead author’s capacity to moderate the focus groups efficiently, and therefore the lead author solely facilitated all remaining focus groups.

The focus group topic guide (Table 3) was continually revised based on reflection and evolving conceptualization. While the core questions remained the same throughout, filter or prompt questions were modified or added as data collection proceeded. For example, while “scale” was a prompt question under “contemporary challenges facing farmers,” this was further refined (scale and financial pressures; scale and workload; scale and farm viability) based on on-going analysis. The topic guide was pilot tested with a small group of dairy farmers (*n* = 3), convened with the help of an advisory group member, prior to full implementation. This resulted in some minor adjustments to the focus group schedule; for example, more attention was given to changing farming policy and its implications for farming practice and farmers’ health with particular consideration for farmers’ stress. All focus groups were recorded using a digital dictaphone. Recordings were manually transcribed verbatim at the earliest possible opportunity after the focus group by the first author and subsequently read by the second author. Data collection ceased once saturation was reached based on Guest et al. (2006, p. 65) definition of saturation “the point in data collection and analysis when new information produces little or no change to the codes, and categories are comprehensively explained so that a theory can emerge from the data.” Field notes and a reflective journal were used to record observations and to contextualize these verbal accounts during transcription and data analysis. For example, field notes were used to document the tone of certain remarks (e.g., sarcasm, humor, anger) to enable the author capture more nuanced and subtle meanings underpinning focus group discussions (Appendix 1). This enabled the lead author to contextualize findings appropriately. All focus groups took place in either a work venue or a local community setting as agreed with each group.

All transcriptions from the focus groups were stored on a password-protected computer and only accessed by the first and second author in accordance with the requirements set out by the General Data Protection Regulation 2018. Audio recordings were transferred to a password protected desktop computer and deleted from the first author’s recording device. All participants were notified that Information would be retained for a further 5 years in a secure environment before being destroyed by deleting files permanently from computers and shredding paper documents.

### Data Analysis

Thematic Content Analysis (TCA) was used to analyse the data. Initially this involved the lead author engaging in “repeated reading” of the data and searching for meaning and patterns (Braun & Clarke, 2006). The next step involved coding the focus group data into categories based on the research questions, breaking the codes into sub-categories, organizing the codes into themes and comparing the themes internally, that is, within a theme, how are participants acting these out (Braun & Clarke, 2006). Themes were identified on patterned responses to the research questions and were decided upon, as Braun and Clarke (2006) suggest, from capturing an important element that the study sought to address. An initial thematic map (Appendix 2) contained five candidate themes. As the analysis progressed it became evident that some of these candidate themes did not have enough data to support them, or did not fit with the research questions, while other candidate themes collapsed into a similar stronger themes. Inter-rater reliability was explored with two transcripts chosen at random on which both the first and second author carried out line-by-line coding. Codes were cross-referenced, discussed, and refined until consensus was reached. All transcripts were then coded iteratively using open and comparative coding techniques, and emerging themes were grouped into parent-themes, sub-themes, and sub-sub-themes (Appendix 4). Following cross-referencing between the first and second author, three parent themes emerged that captured the centrality of the research questions (Appendix 3). Theme memos and notes, and conceptual maps were used to track evolving relationships between themes (Appendix 1, 3, 4). To add to the trustworthiness and rigor of the data collection and analysis process, the study complied key principles outlined by Tolley et al. (2016), namely; (i) credibility—all focus group topics had a logical relationship with the research questions, and were consistent in terms of their explanation; (ii) dependability—the process adopted adhered to the key steps of thematic content analysis outlined by Braun and Clarke (2006); (iii) confirmability—the lead author adopted a reflexive approach in order to make visible personal biases that might have influenced the research process; (IV) transferability—in line with CBPR, the advisory committee had an input to the recruitment of research participants that could provide rich and diverse perspectives on the key research questions.

## Results

The analysis identified three broad themes, and the findings are presented below concerning each of these; “wrestling with challenges to autonomy and control within farming”; “farming masculinities and health”; and “isolation and the decline of rural communities.”

### Wrestling with Challenges to Autonomy and Control Within Farming

A range of distinct challenges emerged for male farmers that were seen as having eroded the degree of autonomy and control they exercised over their farming practices. These included tighter regulatory frameworks associated with changing agricultural policy, pressures associated with scale, and succession and inheritance.

Many farmers highlighted their struggles in adapting to the evolving nature of farming roles that coincided with [common] agricultural policy (CAP) reforms. It was felt that these reforms had changed the nature of farming from a traditional focus on food production to a more administrative role revolving around farm inspections, changes to farm subsidy payments, and scheme paperwork.

*I think farming is kind of gone from your technical knowledge to actually your farm business management. . .and that model is after leaving a lot of farmers behind.* **(FG003, female, 50–59)**

Moreover, farmers felt they were being held to account more and more by what was seen as increasingly punitive regulatory structures. This included living with the threat of being reprimanded or facing sanctions (including financial penalties) should they fail to meet various targets or deadlines—with one farmer questioning “*where does it stop at like*?” **(F008)**. There was also a sense of loss in relation to the autonomy the farmer has with his enterprise. Many felt they were increasingly “losing [their] grip” or control of their farms. Older farmers in particular, were perceived by some, as not having the skills to navigate these new roles, and them, more than most, were being “left behind” by the evolving nature of farming.

*. . .they* [older farmers] *have this fear of Jesus if something’s wrong. . .I’m gonna get thrown under the bus. . .they’re terrified to make any mistake.* **(MNF003, male, 30 - 39)**

Wider concerns about finance were underpinned by the uncertainty and volatility of farming incomes and compounded by what were seen as increasingly paltry and piecemeal financial returns. The change in agricultural policy from a focus on “food security/production” to animal welfare and environmental concerns left many farmers feeling undervalued, and as pawns in a wider power relations contest.

*With all the good intentions of money having to be directed correctly to the right place – there is just no end to it. Before we got a hundred euro for having a suckler cow but now we get twenty euros if we weigh the cow, another twenty euros if we tag the calf, it’s fragmented and it’s very frustrating.* **(F011, male, 30–39)**

Farmers’ frustrations with agri-enviornmental regulations were also deeply rooted in a farming identity that was imbued with tradition, vocation and a deep sense of pride in a particular way of farming. Many felt conflicted by having to conform to these new regulations while simultaneously demonstrating an unwillingness to change and striving to preserve this cultural farming capital, framed around a productivist farming model.

*All this is making life more difficult. They want us to look after the environment along with all the other jobs – but we are not environmentalists, we are tillage and beef farmers and we always were and people before us the same.* **(F010, male, 40–49)**

For some farmers, their decision-making was motivated not just by economic considerations but by core aspects of their identity. For example, while leasing one’s farm might have made financial or work-life balance sense, it was presented in more existentialist terms as emasculating and as a betrayal of farming identity.

*A lot of guys won’t do it* [lease their land] *even though they wouldn’t be making that much. . . And it is because they are associated so much as farmers. You know, you get up and put on the working clothes, and ‘that’s me I am the farmer of the land’.* **(F001, male, 40 - 49)**

The pressures associated with scale—particularly in dairy farming—also emerged as a persistent threat to farmers’ autonomy. Scale coincided with an increase in responsibilities that left many farmers floundering within a culture of “greed” and unmanageable workloads. For many, the extra workload was necessary simply to maintain a basic standard of living.

*There’s greed in it. . .instead of calving 40 cows over 4 months they’re calving 120 over 6 weeks. Farmers are running themselves into the ground trying to keep up.* **(AA015, male, 40–49)**

Many “smaller farmers” faced an uphill task to remain viable, which inevitably led to “double-jobbing,” and fitting their farming work around a full-time job. Sleep deprivation was a significant issue for those farmers. Not surprisingly, it was this constant, more insidious form of chronic financial stress or “cash flow” problems that was more damaging in terms of farmers’ mental health.

*I mean I love farming, but. . .anyone with a small farm and has a family is probably working two jobs and you have very little time for anything else outside working.* **(FG002, female, 50–59)**

*They* [farmers] *are under constant financial pressure. It’s not desperation, or it’s not ‘losing the farm’ kind of pressure; it’s just a constant economic cash flow pressure.* **(P004, female, 50–59)**

Tensions relating to succession and inheritance revolved around younger farmers’ uncertainty and lack of autonomy in not knowing when or if the farm might pass to them. For the farmer “in waiting,” this inevitably led to feeling excluded from key farm decision-making, not having a meaningful income, stagnation, and falling behind one’s [professional] peers; all of which could have debilitating and emasculating effects on the farmer’s self-worth.

*It’s the stagnation. . .you are 20/30 working at home, you don’t know where you stand, you’re making shit money. . .you have no opportunity to stamp your own authority on it.* **(F001, male, 40–49)**

Succession within farming was also deeply imbued with tradition, which further impacted on the incumbent farmer’s autonomy. Tradition dictated that the farm be handed on to “a rightful heir” and that any part thereof not be sold. Inheritance therefore came with a “burden” of expectation surrounding how to engage with the land. Indeed, the prospect of not handing the farm on was associated with a betrayal of one’s responsibility to maintain this tradition.

*My father always worked you know. He didn’t take weekends off, it was just the farm. You feel that responsibility and to some extent the burden of it.* **(F008, male, 60–69)**

For many, inheriting the land also brought additional caring responsibilities. Farmers in the younger age category (20–39) reflected upon the challenge of simultaneously caring for their own children as well as older relatives, while at the same time trying to develop the farm and accumulate savings.

### Farming Masculinities and Health

Farming masculinities in this study were closely aligned with both farming practices and health practices, and were framed in a relational way. The construction of farming masculinities was shaped by working long hours, being committed to hard physical work, and being stoic, self-sufficient, and “strong” in the face of adversity. Indeed, to join the ranks of “tough men” required that farmers’ bodies display these “signs of hard work”—the very essence and embodiment of farming masculinity. This sense of obligation to put their bodies on the line came with the resignation of a cost to their health and to their bodies over time.

*Yeah, I think it’s a pride thing, there are tough men out there in all weathers working hard and have the signs of hard work all over us.* **(F009, male, 30–39)**

By placing their bodies and health in a subservient role to the demands of their farms, many farmers lost sight of the importance of their own health to the welfare of the farm. Ironically, while most saw clear links between animal welfare and farm productivity, the importance of farmers’ own health to the farm’s welfare was more obscure.

*But I don’t think farmers understand their own importance to the farm. Like without them, the farm is nothing. They are the most important cog in the wheel. . .* **(FG003, female, 50–59)**

Indeed, gendered health practices were also framed in a relational way. For example, some [male] farmers proposed that women were overly vigilant or hypochondriac about health issues and were, therefore, an inappropriate yardstick against which to gauge how men should care for their health. This was reinforced further by both male and female perceptions of women as gatekeepers or custodians of men’s health.

*But compared to women though, you wouldn’t go* [to a GP] *a fraction of the time, sure they are always going.* **(F002, male, 40–49)**

*It took me about three years to basically bully my husband into going for a medical, you’d have to come up with all of these excuses.***(FG004**, **female, 50–59**)

Prevailing farming masculinity norms frequently militated against farmers asking for help or support during times of ill-health or distress, but rather their “in-built” default position was to endure hardship and “soldier on.” Farmers’ reluctance to seek help was particularly prevalent regarding mental health. To show vulnerability was seen as an admission of failure that would not be looked on favourably in a tight-knit rural community.

*I would say the old perception of ‘we are hardy and we are men, and we are ok. We don’t have problems’. That’s the perception.* **(F003, male, 50–59)**

*The neighbour thing is a big problem also. Farmers are wondering ‘what will the neighbours think of this?’ You don’t want the community knowing about it.* **(AA011, male, 20–29)**

Farmers’ approach to help-seeking was shaped by two critical aspects of their professional practice; the ability to problem-solve and to draw on a multi-disciplinary skillset to solve problems. Farmers handling of agricultural problems essentially framed their approach to “fixing” health problems—to “outsource” help for a health problem, only after drawing on all of one’s own resources to solve the matter. Only then was it seen as acceptable to seek help. To acknowledge vulnerability by seeking help was seen as an affront to one’s capacity to care for one’s farm (and one’s family) and to be a “fixer.”

*. . .he* [farmer] *works on his own and he is used to problem-solving, so he is multi-disciplined, whereas you go into another occupation they are trained in one stream of work, and if they require help, they outsource it. You’re very self-sufficient as a farmer, and the thing is to ask for help when you require it.* **(F009, male, 30–39)**

For farmers in the older age categories in particular, there was, paradoxically, the fear of finding out something was wrong, or opening up a “can of worms”—the safer option being to “leave well enough alone.” However, other farmers’ experiences suggested that cultural norms associated with health and help-seeking were not fixed and that given the right environment, where farmers could share and talk openly about common problems, the “weight” of a problem could be lessened. The experience of a health crisis, either personally or through a loved one, also prompted a more proactive approach.

*My father died of a heart-attack at 44 and I knew when I came to 43, I was getting my heart checked out. . .You are putting an awful lot at risk by not simply going once in a while.* **(F009, male, 30–39)**

### Isolation and the Demise of Rural Communities

Rural isolation was a recurring theme across all transcripts. The demise of rural communities more generally coupled with increased loneliness associated with reduced social interaction, were seen as having left more “at risk” single and older farmers particularly vulnerable. There were particular concerns about what was seen as the unraveling of rural communities; underpinned by a wider backdrop of a decline in the number of viable farm holdings, migration of rural dwellers to urban areas for work, and more limited opportunities for social gatherings (closure of rural shops and pubs). Being disconnected from human contact was seen as inhibiting the development of meaningful conversations that could sustain relationships. An important backdrop to these findings was the belief that the traditional farming “meitheal” (mutual support and communal way of working) and the passing on skills between generations was also being lost.

*. . .you don’t actually meet enough people to develop the conversation around things.* **(F006, male, 60–69)**

*I have seen a marked change in rural society. . .help[ing] your neighbor out; that has really suffered.* **(F0013, male, 50–59)**

Underpinning rural isolation was the perception of having been let down or abandoned by the key pillars of civil society. For example, on the question of security and rural break-ins, there was a perception of rural Ireland being “*left* [to] *fend for itself*” **(F004)**. Thus, at the core of rural isolation was a lack of control or agency, which with the onset of age saw a deepening of vulnerability and perception of abandonment. Notably, some farmers felt neglected and let down by their own farmer representative bodies whose primary role was to represent their interests. For example, participants in the West of Ireland focus group expressed anger at what they perceived as the failure of farming authorities to appreciate or act upon the unique challenges that were characteristic of their farming land.

*Now I remember saying to (senior farming official). . .‘You put a pair of waders on, and you come down to my farm. . .and you’ll know what we in the West of Ireland want. . . you’ll see the difference in where you are and where we are’.* **(P006, female, 40–49)**

The isolated nature of farming and the sheer invisibility of farmers relative to other socially interactive occupations was a recurring concern among participants. Older and middle-aged single farmers living with an elderly parent were identified as being particularly vulnerable in terms of isolation and loneliness. Additionally, some farmers were seen as being “at risk” of being institutionalized by farming; consumed and locked into their routine of farming work.

*A man in his 40s or 50s still living with his mother, you have a man that is definitely in trouble and needs help.***(AC005**, **female, 60–69**)

Isolation was also associated with a loss of perspective, as the isolated farmer did not have anyone or anywhere to discuss or unburden his or her difficulties. This was compounded by more entrenched masculine norms which militated against farmers seeking help—hence, problems did not just remain unresolved, but rather intensified, festered and became amplified over time. This ran the risk of farmers dwelling on and apportioning blame to themselves for problems and questioning their own self-worth as farmers. Farmers, who were more socially engaged, on the other hand, were seen as being more resilient.

*I think for the more isolated farmer, there is just no degree of perspective, where there is a problem on the farm. For instance, if a calf dies, they are ramming that around their heads all day; whereas* [for] *someone else, that is forgotten about by 11 o’clock.* **(AC003, male, 40–49)**

Indeed, it was felt that the ripple effects of isolation that prompted a deeper sense of hopelessness and despair for many in rural communities that had the potential to cascade into more pressing mental health problems. There was a perception that the farmer who was both isolated and had underlying mental health problems was the most difficult to engage.

*Country people in general, their mental health has dropped, and that’s because of isolation it has developed into mental illness. People are not out. No engagement at all.* **(F007, male, 50–59)**

## Discussion

This study was commissioned to inform the design of a bespoke farmers’ health training program to enable agricultural advisors in Ireland to play a more proactive role in supporting male farmers on health issues. Against a backdrop of poor health outcomes more generally among male farmers as well as substantial changes in farming governance, this study explored how contemporary challenges and stressors currently facing farmers, within an Irish context, impact on farmers’ identities, masculinities and health, and examined the ripple effects for rural communities. The focus of the study therefore was not to investigate specific health issues impacting male farmers; rather it was hoped that findings would provide rich insights into both the lived experiences of male farmers in Ireland as well as garnering the perspectives of key informants on the issues impacting on male farmers’ health, that would serve as an important backdrop to the design of the proposed farmers’ health training program.

In the context of research question one, findings indicate that farmers in Ireland are confronted by a range of distinct challenges that negatively impact the degree of autonomy and control they currently exercise over their farm enterprises. Chief among these are changing farming roles and being held to account by what they perceive are regimented and punitive regulatory structures; the increase in responsibilities and financial pressures associated with scale; as well as, for younger farmers, the procrastination, uncertainty, burden of expectation and additional caring responsibilities that are associated with succession. These tensions were exacerbated by changes associated with the restructuring of agricultural policy and structural features of farming / rural society, such as concern for what neighbors might think or say. This latter point is reflected in international and Irish studies that have found that farmers can be judgmental of their counterparts (Ní Laoire, 2005; Sutherland & Burton, 2011). Tensions ran exceptionally high among those farmers who felt torn between more traditional endeavors to acquire cultural farming capital - framed around a productivist farming model - and the modern requirement to conform to agri-enviornmental regulation standards. This jarred with farmers in two critical ways. Firstly, farmers’ new role as custodians of the natural landscape and environment disrupted deeply entrenched notions of ‘the good farmer’ that constructed farmers’ sense of self-worth and status within the community as a product of the intensification of production (Burton et al., 2008). Secondly, it marked a particularly challenging transition for many farmers as they sought to retain a degree of autonomy and control in the face of increasing regulation and inspection (Forney, 2016).

Previous studies within an Irish context have highlighted the “burden of expectation” associated with succession (Cassidy, 2017). Factors associated with scale such as high workload, financial pressure and limited social support were pre-cursors for mental and emotional distress (Furey et al., 2016). It is also well established that, for those farmers whose identity is heavily invested in their farming, changes that threaten or undermine that role can be particularly challenging. For example, a UK study found that where male farmers’ identity was deeply rooted in the cultural and physical spaces of farming, rates of suicide increased when their livelihood became threatened, and they could not imagine a way of being other than “the farmer” (O’Hagan, 2001). Findings from this study emphasize the importance of accounting for the ripple effects on farmers’ health and wellbeing when considering wider changes to agricultural policy and restructuring.

In the context of research question two, the concept of “the good farmer” also had parallel links with the construction of farming masculinities as described in this study. Having a strong work ethic and an appetite for hard physical work, and being stoic, self-sufficient and “strong” in the face of adversity, were the active ingredients that shaped more dominant or hegemonic farming masculinities—characteristics that are consistent with previous findings (Ní Laoire, 2005; Roy et al., 2017). Farming masculinities in this study were deeply embedded in gender relations and farming tradition—framed within the socio-cultural context of tight-knit rural communities. They were conceptualized as being distinct from what was regarded as the more feminine domain of health. Previous studies have noted how the intensification of farming practices and increased mechanization that accompanied the rise in productivist agriculture represented the new masculinization of farming (Brandth, 1994). Similarly, resistance to more recent agri-environmental policy measures also has strong links to the construction of farming masculinities (Peter et al., 2009). For example, a U.S. study found that farmers’ resistance to agri-environmental policy measures was underpinned by “monologic” masculinity—characterized by rigid and strictly negotiated performance outcomes rooted in controlling nature to facilitate productivist agricultural methods (Peter et al., 2009). This would suggest that beneath the valorized methods of acquiring cultural capital among farmers is a more profound association with masculine norms of autonomy and dominance. By contrast, Peter et al.’s (2009) notion of “dialogic” masculinity was associated with less need for control over nature and greater compliance with agri-environmental measures. Findings from this study add to a significant body of literature on the concept of the ‘the good farmer’ (Burton et al., 2021) and its implications for how farmers relate to health. Furthermore, we argue that an important starting point for any future endeavors to promote farmers’ health must be to contest the notion of “the good farmer” in order to facilitate a cultural shift that makes it more acceptable for farmers to engage in health.

Findings from this study revealed that the pursuit of hegemonic farming masculinity had ripple effects on farmers’ health and farmers’ bodies. Indeed the visible “signs of hard work,” which manifested as wear and tear on farmers’ bodies, was a badge of honor that embodied this masculine ideal. Inevitably, this conflicted with any proposition to prioritize health needs or to show vulnerability and seek help. For most farmers in this study, health was seen as “women’s business,” with feminine connotations, and not something “hard-working men” had time to engage with (Roy & Hočevar, 2019). Notably, some farmers contested such framings, extolling the virtues of a more responsible and proactive approach to health over more stoic and reactive approaches. Wenger (2011) notes that men’s health engagements operate more on a pathway rather than as a binary, single decision leading toward or away from health services. We argue that more oncerted efforts are needed to promote an increased focus on farmers’ health within Ireland that contest the essentially unhealthy nature of the more dominant or hegemonic form of farming masculinity, and that facilitate more farmers to move along the pathway to improved self-care. From a gender relations and a gender equality perspective, such efforts are also warranted to achieve a more equitable distribution of caring and nurturing responsibilities in farming households (Byrne et al., 2013; Shortall et al., 2017).

The act of seeking help for a mental health issue posed particular challenges for farmers in this study and ran the risk of being consigned to a subordinate masculine status within one’s community. Previous studies have shown how male farmers tend to equate health practices with femininity (Ní Laoire, 2005; Roy et al., 2017) and that help-seeking, in particular, can be seen as compromising their allegiance to masculine norms of independence and stoicism (Gast & Peak, 2011; Roy et al., 2017). However, avoidance of help-seeking can escalate to more pressing health problems, particularly mental health problems (Kennedy et al., 2020; Roy et al., 2013). A notable finding was a tendency by some farmers to count on what they saw as their multi-disciplinary farming skillset to “fix” health problems—an option that, for some, was more appealing than admitting “failure” or “opening a can of worms” by deferring to medical expertise. This finding is consistent with how men engaged in labor-intensive work and frequently adopt a more functional and mechanistic conceptualization of health (Watson, 2000). Nonetheless, it should be acknowledged that farmers’ help-seeking behaviors in this study were not static. For example, in response to a “wake-up call,” some farmers were open to adopting a more pragmatic and proactive stance on health issues. Previous studies have shown that experiencing a health crisis (either personally or through a loved one) can prompt a heightened sense of responsibility for health among men (Richardson, 2010). Similarly, increasing awareness of their vulnerability to disease with age has been highlighted as a catalyst for positive health behavior among older men (Gough & Conner, 2006; Peak & Gast, 2014). These findings therefore add to existing literature (Courtenay, 2011; Roy et al., 2014) by highlighting how rural masculine practices are fluid, rather than fixed or static, and demonstrate that, with appropriate supports, farmers might be open to more actively engaging in their health.

In the context of research question three, grave concerns were expressed about what was seen as the unraveling of rural communities, underpinned in particular, by the decline in the number of viable farm holdings, the closure of rural services and social outlets, the loss of community spirit or “meitheal,” and by a sense of having been let down or abandoned by the key pillars of the state. Moreover, this increased the risk of isolation and loneliness and the “festering” of problems over time, with single farmers caring for an elderly parent being seen as particularly vulnerable. These findings are consistent with previous studies that highlight a change in farm succession and more solitary farming practices (Cassidy, 2017; Price & Evans, 2009), resulting in a pattern of diminishing social circles for farmers and inevitably leading to an increase in loneliness, with ripple adverse effects on farmers’ well-being (Kennedy et al., 2020; Monk, 2000; Roy et al., 2014). Chronic social isolation has been identified as a high-risk factor for loneliness and poor mental and physical health (Carroll et al., 2014; Verdonk et al., 2010). Loneliness has been attributed to deficits in relationships from intimate partner to wider community levels, with far-reaching costs to the individual (Cacioppo & Hawkley, 2009). Parallel measures that seek to combat rural isolation and to restore the social fabric of rural communities are critically important for greater social engagements among the farming community. Against the broader backdrop of multiple challenges and stressors in farmers’ lives highlighted in this study, the stress process theory (Pearlin et al., 1981) provides a useful framework for any future attempts to tackle the sources, mediators and manifestations of stress in farmers.

## Conclusion

This study’s findings provide important insights into the contemporary challenges and stressors farmers experience and, in particular, how the evolving nature of these stressors as well as changes in farming governance have impacted on farmers’ identities, masculinities, and health. Tensions were particularly evident among those farmers whose ideals and allegiances were still based on “the good farmer” and on a productivist agricultural model, but who were increasingly being constrained and held to account by agri-environmental regulations. These tensions posed particular challenges for farmers’ mental health. In particular, the valorized pursuit of a “strong” masculine identity was at odds with male farmers’ willingness or motivation to seek help. Notably, not all male farmers were resistant to caring for their health, highlighting the fluidity of masculinities and, as highlighted by Wenger (2011), men’s relationship with health and help-seeking is a “*trajectory or pathway, rather than an isolated, single decision point leading toward or away from medical services.*”

Our findings raise several important implications in terms of informing the design of a farmers’ health training program. The program needs to be situated within a socio-ecological model (World Health Organization, 2012, 2014) of farmers’ health that accounts in particular for how farming identity and masculinities are shaped by economic, environmental and socio-cultural factors within a dynamic and evolving rural and farming context. It must also be acknowledged that farmers are not a homogenous group and that diversity in terms of age, gender, geography, marital status, caring responsibilities, enterprise and seasonal factors, predispose different sub-groups of farmers to different health challenges. However, the proposed program cannot be seen as a panacea for all farmers’ ills, but rather as one component of a broader suite of measures—at a policy, advocacy, education and training, and service delivery level—that seeks to cohesively address farmers’ health issues. At a practice level, findings clearly demonstrate that safe spaces are needed for farmers to contest more restrictive farming masculinities and notions of “the good farmer” to address their resistant behavior to prioritizing their own health needs. Specific support measures are needed to address the needs of more vulnerable and isolated farmers, particularly from a suicide prevention perspective. Finally, parallel measures that seek to combat rural isolation and restore the social fabric of rural communities are critically important in optimizing the utility of the proposed program in tackling the issue loneliness among farmers.

## Limitations

While this study aimed to seek the views of a diverse sample, the findings cannot purport to represent the views of all target groups. The lead researcher’s positionality must also be taken into account when interpreting the findings of this study. As a young, healthy male in a position of authority within the focus groups, he may have been perceived as an “expert” with the expectation of participants to divulge information. Additionally, while all research participants were assured of confidentiality and invited to speak candidly, some may have been guarded in sharing their experiences. The study would have been enhanced by the inclusion of non-conventional farmers (e.g., organic farmers) and more participants who engage with farmers regularly, that is, mart managers, co-op managers, agri-business and finance officials. Three part-time farmers were part of the advisory group—on reflection, the inclusion of at least one full-time farmer would have been beneficial and in keeping with the spirit of CBPR. When considering the qualitative rigor of the study, the analysis of primary themes and sub-themes would have been heightened through a third reviewer. Also in terms of qualitative rigor (Tolley et al., 2016), particular limitations may lie in the transferability of the research findings to the wider population of male farmers, with only 13 farmers represented among the three male farming focus groups. However, the “key informant” focus groups give a broader contextual voice to the farming population with respect to the research questions. Whist focus groups represented diversity in terms of geographical location, age-profile, gender and farming enterprise, the overall number of participants represented across all focus groups was a limitation. Future research should increase the breadth of research participants, and cross-reference research findings with similar populations in other countries. Future research should also focus on the specific challenges and stresses impacting on female farmers.

## Figures and Tables

**Appendix 2.**
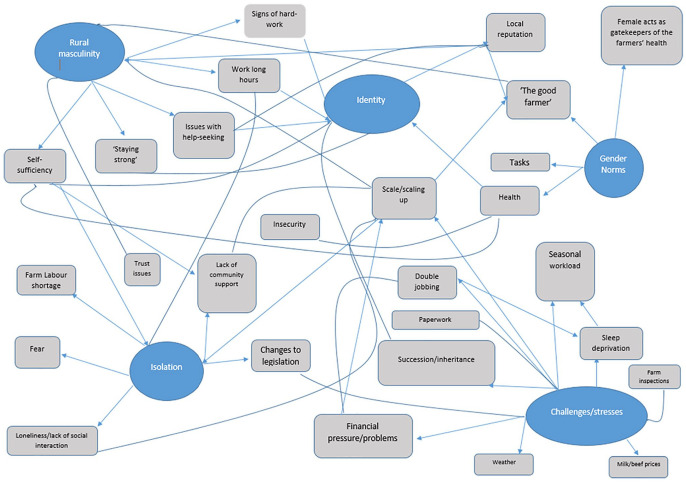
Initial Concept Map Containing Primary Themes and Sub-themes

**Appendix 3.**
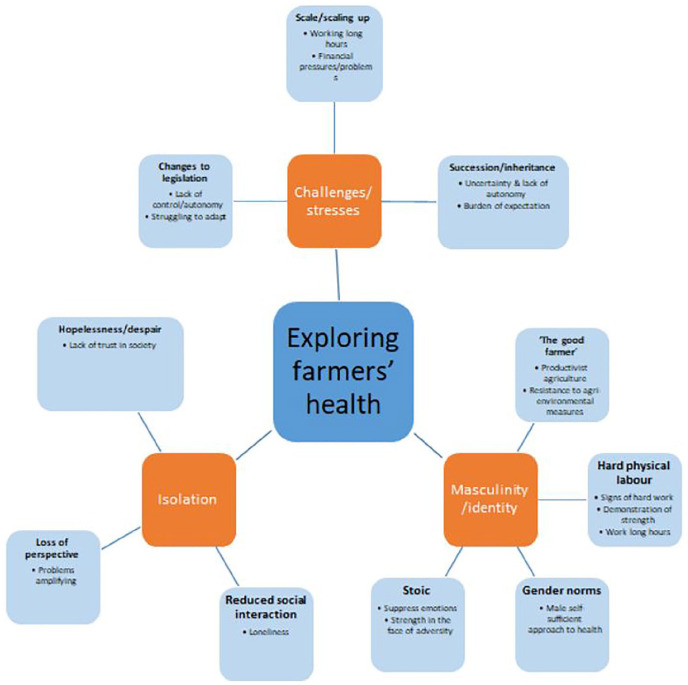
Final Concept Map Used for This Study *Following cross-reference from the first and second author, three parent themes emerged that captured the centrality of the research questions.

## Appendices

### Appendix 1: Sample of Memos and Notes Used from Focus Groups

*But compared to women though you wouldn’t go [to GP] a fraction of the time, sure they are always going.* **F002**

- Said flippantly and with a sense of humor. Other participants laughing gently

*To us now, in case we weren’t looking after our selves (yeah, yeah). So our farmers are self-employed. We have to do it (we have to do it) . . .that’s another thing then. (Yeah yeah) Teagasc are looking after the lads out there. . .who is looking after us? (Exactly, yea).* **F008**

- Said with a sense of despair and frustration

*Well, look. We’ve known for a long time that unmarried men don’t live as long as married men. So, do you put in place that every fella should get married?* **F009**

- laughter among participants, followed by jokes to each other.

*Because you know why. Modernization, technology and modern farming methods – all of them things have left farmers like us behind. I love farming but my farming isn’t valued anymore, nobody cares about us. Before you could make a good living of having cattle but not anymore.* **F013**

- Said with sadness and despair, and what seemed to be an element of embarrassment in front of peers

*Not an area* (health*) id be very strong in anyway but I’ll do my best (laughs)* **AA004**

- Said in jest and other participants laughing

**Appendix 4.** Sample Technique Used to Identify Themes (Braun & Clarke, 2006).

**Parent theme**	**Challenges/stresses**	**Masculinity/identity**	**Isolation**
**Sub theme**	Changes to legislation	The good farmer	Reduced social interaction
Sub-sub theme	-lack of control/autonomy -Struggling to adapt	-productivist -resistance to agri-evnironmental	-loneliness
Data extracted	*. . .they* [older farmers] *have this fear of Jesus if something’s wrong. . .I’m gonna get thrown under the bus. . .they’re terrified to make any mistake.* **(MNF003, male)**	*They want us to look after the environment along with all the other jobs – but we are not environmentalists, we are tillage and beef farmers and we always were and people before us the same.* **(F010, male)**	*. . .you don’t actually meet enough people to develop the conversation around things.* **(F006, male)**
**Sub theme**	Scale/scaling up	Physical labor	Loss of perspective
Sub-sub theme	-Working long hours -Financial pressure/problems	-signs of hard work -demonstration of strength -work long hours	-Problems amplify
Data extracted	*There’s greed in it. . .instead of calving 40 cows over 4 months they’re calving 120 over 6 weeks. Farmers are running themselves into the ground trying to keep up.* **(AA015, male)**	*Yeah, I think it’s a pride thing, there are tough men out there in all weathers working hard and have the signs of hard work all over us.* **(F009, male)**	*for the more isolated farmer, there is just no degree of perspective, where there is a problem on the farm. For instance, if a calf dies, they are ramming that around their heads all day; whereas* [for] *someone else, that is forgotten about by 11 o’clock.* **(AC003, male)**
**Sub theme**	Succession/inheritance	Gender norms	Hopelessness/despair
Sub-sub them	-uncertainty and lack of autonomy -burden of expectation	-Self-sufficient approach to health -Provider	-Lack of trust in society
Data extracted	*It’s the stagnation. . .you are 20/30 working at home, you don’t know where you stand, you’re making shit money. . .you have no opportunity to stamp your own authority on it.* **(F001, male)**	*he* [farmer] *works on his own and he is used to problem-solving. . .You’re very self-sufficient as a farmer, and the thing is to ask for help when you require it.* **(F009, male)**	*It’s a waste of time telling the guards anyway. What are they going to do?*(re a previous break-in) **(F008, male)**
**Sub theme**		Stoic	
Sub-sub theme		-Suppress emotions -strength in the face of adversity	
Data extracted		*It’s not in our generation to admit you know, Jaysus I am stressed, you know like (yea, yea) [21.07]* **(F006, male)** *It’s like admitting you are a failure more or less* **(F006, male)**	

## Notes

1 Farming that is associated with livestock to produce milk products

2 Farming that is associated with livestock to produce meat products

3 Farming that is associated with cultivating the soil to produce crops

4 Voluntary organization representing young farmers

## References

[bibr1-15579883211035241] Amshoff S. K. Reed D. B. (2005). Health, work, and safety of farmers ages 50 and older. Geriatric Nursing, 26(5), 304–308. 10.1016/j.gerinurse.2005.08.008 16213982

[bibr2-15579883211035241] Baum F. Macdougall C. Smith D. (2006). Participatory action research. Journal of Epidemiology and Community Health, 60(10), 854–857. 10.1136/jech.2004.028662 16973531 PMC2566051

[bibr3-15579883211035241] Bloom D. E. Cafiero E. Jané-Llopis E. Abrahams-Gessel S. Bloom L. R. Fathima S. Feigl A. B. Gaziano T. Hamandi A. Mowafi M. Pandya A. Prettner K. Rosenberg L. Seligman B. Stein A. Z. Weinstein C. (2011). The Global Economic Burden of Noncommunicable Diseases (Issue September). http://www3.weforum.org/docs/WEF_Harvard_HE_GlobalEconomicBurdenNonCommunicableDiseases_2011.pdf

[bibr4-15579883211035241] Brandth B. (1994). Changing femininity the social construction of women farmers in Norway. Sociologia Ruralis, 34(2–3), 127–149.

[bibr5-15579883211035241] Braun V. Clarke V. (2006). Using thematic analysis in psychology. Qualitative Research in Psychology, 3(2), 77–101. 10.1191/1478088706qp063oa

[bibr6-15579883211035241] Burns L. (2021). Challenges to habitus: Scruffy hedges and weeds in the irish Countryside. Sociologia Ruralis, 61(1), 2–25. 10.1111/soru.12307

[bibr7-15579883211035241] Burton R. (2004). Seeing through the ‘Good Farmer’s’’ eyes : Toward developing an understanding of the social symbolic value of “productivist ” behaviour.’ Sociologia Ruralis, 44(2), 195–215.

[bibr8-15579883211035241] Burton R. Forney J. Stock P. Sutherland L.-A. (2021). The good farmer: Culture and identity in food and agriculture. Earthscan from Routledge. https://books.google.ie/books?hl=en&lr=&id=zhj0DwAAQBAJ&oi=fnd&pg=PT9&dq=burton+The+good+farmer&ots=-cBqciy6oU&sig=-aXVlJk4uHyaD741IEYqqIakI4U&redir_esc=y#v=onepage&q=burtonThegoodfarmer&f=false

[bibr9-15579883211035241] Burton R. Kuczera C. Schwarz G. (2008). Exploring farmers’ cultural resistance to voluntary agri-environmental schemes. Sociologia Ruralis, 48(1), 16–37. 10.1111/j.1467-9523.2008.00452.x

[bibr10-15579883211035241] Burton R. Wilson G. A. (2006). Injecting social psychology theory into conceptualisations of agricultural agency: Towards a post-productivist farmer self-identity? Journal of Rural Studies, 22, 95–115. 10.1016/j.jrurstud.2005.07.004

[bibr11-15579883211035241] Byrne A. Duvvury N. Macken-Walsh Á. Watson T. (2013). Gender, power and property: In my own right. The Rural Economy Development Programme (REDP) Working Paper Series.

[bibr12-15579883211035241] Cacioppo J. T. (2011). Social neuroscience. Why loneliness is hazardous to your health. Science (New York, N.Y.), 331(January), 138–140. 10.1126/science.331.6014.138 21233358

[bibr13-15579883211035241] Cacioppo J. T. Fowler J. H. Christakis N. A. (2009). Alone in the crowd: The structure and spread of loneliness in a large social network. Journal of Personality and Social Psychology, 97(6), 977–991. 10.1037/a0016076 19968414 PMC2792572

[bibr14-15579883211035241] Cacioppo J. T. Hawkley L. C. (2009). Perceived social isolation and cognition. Trends in Cognitive Sciences, 13(10), 447–454. 10.1016/j.tics.2009.06.005 19726219 PMC2752489

[bibr15-15579883211035241] Carroll P. Kirwan L. Lambe B. (2014). Engaging hard to reach men in community based health promotions. International Journal of Health Promotion and Education, 52(3), 120–130. 10.1080/14635240.2013.876185

[bibr16-15579883211035241] Carroll P. Noel Richardson N. Grace B. (2020). Connecting with young men’: The impact of training in ireland. International Journal of Men’s Social and Community Health, 3(1), e24–e35. 10.22374/ijmsch.v3i1.26

[bibr17-15579883211035241] Cassidy A. (2014). ‘I’m not going to be able to leave’: The impact of belonging to the Irish farming community on university students’ life experiences and transitions to adulthood. PhD Thesis. NUI - Galway.

[bibr18-15579883211035241] Cassidy A. (2017). ‘I like it – I just don’t know what to do with it.’: The student-successor in Irish family farming. Irish Geography, 50(2), 193–208. 10.2014/igj.v50i2.1322

[bibr19-15579883211035241] Cleary A. (2005). Death rather than disclosure: Struggling to be a real man. Irish Journal of Sociology, 14(2), 155–176.

[bibr20-15579883211035241] Cleary A. (2012). Social science & medicine suicidal action, emotional expression, and the performance of masculinities. Social Science & Medicine, 74(4), 498–505. 10.1016/j.socscimed.2011.08.002 21930333

[bibr21-15579883211035241] Connell R. (1995). Masculinities. Policy Press. https://books.google.ie/books?hl=en&lr=&id=YuR2uFxxvPoC&oi=fnd&pg=PR5&dq=connell+1995+masculinities+pdf&ots=GycTLgNs0W&sig=Pd8Jsq24R7ISqt_fGQBXzU6LV6I&redir_esc=y#v=onepage&q&f=false

[bibr22-15579883211035241] Courtenay W. H. (2000). Constructions of masculinity and their influence on men’s well-being: A theory of gender and health. Social Science & Medicine, 50, 1385–1401. http://www.postpartummen.com/pdfs/SS&M.PDF 10741575 10.1016/s0277-9536(99)00390-1

[bibr23-15579883211035241] Courtenay W. H. (2011). Dying to be men: Psychosocial, environmental, and biobehavioral directions in promoting the health of men and boys. (1st ed.). Routledge/Taylor & Francis Group. https://www.routledge.com/Dying-to-be-Men-Psychosocial-Environmental-and-Biobehavioral-Directions/Courtenay/p/book/9780415878760

[bibr24-15579883211035241] CSO. (2016). Women and men in Ireland 2016 - CSO - Central Statistics Office. https://www.cso.ie/en/releasesandpublications/ep/p-wamii/womenandmeninireland2016/

[bibr25-15579883211035241] Cush P. Macken-Walsh Á. (2018). Reconstructing male identities through joint farming ventures in Ireland. Sociologia Ruralis, 58(4), 726–744. 10.1111/soru.12212

[bibr26-15579883211035241] Department of Agriculture, Food and the Marine (2000). CAP explained: Department of agriculture, food & the marine. Department of Agriculture, Food & the Marine.

[bibr27-15579883211035241] Forney J. (2016). Blind spots in agri-environmental governance: Some reflections and suggestions from Switzerland. Review of Agricultural, Food and Environmental Studies, 97, 1–13. 10.1007/s41130-016-0017-2

[bibr28-15579883211035241] Fraser C. E. Smith K. B. Judd F. Humphreys J. S. Fragar L. J. Henderson A. (2005). Farming and mental health problems and mental illness. International Journal of Social Psychiatry, 51(4), 340–349. 10.1177/0020764005060844 16400909

[bibr29-15579883211035241] Furey E. M. O’Hora D. McNamara J. Kinsella S. Noone C. (2016). The roles of financial threat, social support, work stress, and mental distress in dairy farmers’ expectations of injury. Frontiers in Public Health, 4(June), 1–11. 10.3389/fpubh.2016.00126 27446893 PMC4914495

[bibr30-15579883211035241] Gallagher A. G. Sheehy N. P. (1994). Suicide in rural communities. Journal of Community & Applied Social Psychology, 4(3), 145–155. 10.1002/casp.2450040302

[bibr31-15579883211035241] Gast J. Peak T. (2011). “It used to be that if it weren’t broken and bleeding profusely, i would never go to the doctor”: Men, masculinity, and health. American Journal of Men’s Health, 5(4), 318–331. 10.1177/1557988310377926 20798142

[bibr32-15579883211035241] Gough B. Conner M. T. (2006). Barriers to healthy eating amongst men: A qualitative analysis. Social Science and Medicine, 62(2), 387–395. 10.1016/j.socscimed.2005.05.032 16011867

[bibr33-15579883211035241] Guest G. Bunce A. Johnson L. (2006). How many interviews are enough?: An experiment with data saturation and variability. Field Methods, 18(1), 59–82. 10.1177/1525822X05279903

[bibr34-15579883211035241] Health and Safety Authority (HSA). (2015). Work-related stress a guide for employers. In Health & safety authority. http://www.hsa.ie/eng/Publications_and_Forms/Publications/Occupational_Health/Work_Related_Stress_A_Guide_for_Employers.pdf

[bibr35-15579883211035241] Hennessy T. Kinsella A. (2013). 40 years of Irish farming since joining the European Union: A journey with the Teagasc National Farm Survey 1972 to 2012.

[bibr36-15579883211035241] Israel B. A. Schulz A. J. Parker E. A. Becker A. B. (2008). Critical issues in developing and following community-based participatory research principles (pp. 47–62). Jossey-Bass. https://www.scholars.northwestern.edu/en/publications/critical-issues-in-developing-and-following-community-based-parti-2

[bibr37-15579883211035241] Johnson B. Clarke J. M. Johnson B. (2003). Collecting sensitive data. Qualitative Health Research, 13(3), 421–434. 10.1177/1049732302250340 12669341

[bibr38-15579883211035241] Jull J. Giles A. Graham I. D. (2017). Community-based participatory research and integrated knowledge translation: Advancing the co-creation of knowledge. Journal of Implementation Science, 12(150), 1–9. 10.1186/s13012-017-0696-3 29258551 PMC5735911

[bibr39-15579883211035241] Kennedy A. Adams J. Dwyer J. Rahman M. A. Brumby S. (2020). Suicide in rural Australia: Are farming-related suicides different? International Journal of Environmental Research and Public Health, 17(6). 10.3390/ijerph17062010 PMC7143525 32197446

[bibr40-15579883211035241] Layte R. Banks J. Walsh C. McKnight G. (2015). Trends in socio-economic inequalities in mortality by sex in Ireland from the 1980s to the 2000s. Irish Journal of Medical Science, 184(3), 613–621. 10.1007/s11845-014-1189-x 25156180

[bibr41-15579883211035241] Lefkowich M. Richardson N. Brennan L. Lambe B. Carroll P. (2018). A process evaluation of a Training of Trainers (TOT) model of men’s health training. Health Promotion International, 33(1), 60–70. 10.1093/heapro/daw056 27476866

[bibr42-15579883211035241] Lefkowich M. Richardson N. Robertson S. (2017). “If we want to get men in, then we need to ask men what they want”: Pathways to effective health programing for men. American Journal of Men’s Health, 11(5), 1512–1524. 10.1177/1557988315617825 PMC5675200 26614444

[bibr43-15579883211035241] Letnes J. M. Torske M. O. Hilt B. Bjørngaard J. H. Krokstad S. (2016). Symptoms of depression and all-cause mortality in farmers, a cohort study: The HUNT study, Norway. BMJ Open, 6(5), 1–9. 10.1136/bmjopen-2015-010783 PMC4874135 27188811

[bibr44-15579883211035241] Lunner Kolstrup C. Kallioniemi M. Lundqvist P. Kymäläinen H. R. Stallones L. Brumby S. (2013). International perspectives on psychosocial working conditions, mental health, and stress of dairy farm operators. Journal of Agromedicine, 18(3), 244–255. 10.1080/1059924X.2013.796903 23844791

[bibr45-15579883211035241] Mahalik J. R. Locke B. D. Ludlow L. H. Diemer M. A. Scott R. P. J. Gottfried M. Freitas G. (2003). Development of the conformity to masculine norms inventory. Psychology of Men & Masculinity, 4(1), 3–25. 10.1037/1524-9220.4.1.3

[bibr46-15579883211035241] Masadeh M. A. (2012). Focus group: Reviews and practices focus group: Reviews and practices Al-Hussein Bin Talal University, Petra college for tourism and archaeology. International Journal of Applied Science and Technology, 2(10), 63–68.

[bibr47-15579883211035241] Mcintosh W. L. Spies E. Stone D. M. Lokey N. Trudeau T. Bartholow B. (2016). Morbidity and mortality weekly report suicide rates by occupational group-17 States, 2012. http://www.cdc.gov/mmwr/cme/conted_info.html#weekly. 10.15585/mmwr.mm6525a1 27359167

[bibr48-15579883211035241] Minnotte K. L. Yucel D. (2018). Work–family conflict, job insecurity, and health outcomes among US workers. Social Indicators Research, 139(2), 517–540. 10.1007/s11205-017-1716-z

[bibr49-15579883211035241] Monk A. (2000). The influence of isolation on stress and suicide in rural areas: An international comparison. Rural Society, 10(3), 393–403. 10.5172/rsj.10.3.393

[bibr50-15579883211035241] Morgan D. (2014). Successful focus groups: Advancing the state of the art. In Successful focus groups: Advancing the state of the art. SAGE Publications, Inc. 10.4135/9781483349008

[bibr51-15579883211035241] Murthy V. (2020). Together (First). Profile Books Ltd.

[bibr52-15579883211035241] NASEM. (2020). Social isolation and loneliness in older adults: Opportunities for the healthcare system (First). The National Academic Press. 32510896

[bibr53-15579883211035241] Ní Laoire C. (2005). “You’re not a man at all!”: Masculinity, responsibility, and staying on the land in contemporary Ireland. Irish Journal of Sociology, 14(2), 94–114.

[bibr54-15579883211035241] O’Brien R. Hunt K. Hart G. (2005). “It’s caveman stuff, but that is to a certain extent how guys still operate”: Men’s accounts of masculinity and help seeking. Social Science and Medicine, 61(3), 503–516. 10.1016/j.socscimed.2004.12.008 15899311

[bibr55-15579883211035241] O’Donnell S. Richardson N. (2018). Middle-aged men and suicide in Ireland. Health Service Executive (HSE), Ireland.

[bibr56-15579883211035241] O’Hagan A. (2001). The end of British farming. Profile Books Ltd. https://www.abebooks.co.uk/signed-first-edition/End-British-Farming-OHagan-Andrew-London/1169381967/bd

[bibr57-15579883211035241] Osborne A. Carroll P. Richardson N. Doheny M. Brennan L. Lambe B. (2018). From training to practice: The impact of ENGAGE, Ireland’s national men’s health training programme. Health Promotion International, 33(3), 458–467. 10.1093/heapro/daw100 28013256

[bibr58-15579883211035241] Peak T. Gast J. A. (2014). Aging men’s health-related behaviors. Sage Open, 4(4), 1–10. 10.1177/2158244014558044

[bibr59-15579883211035241] Pearlin L. I. Menaghan E. G. Lieberman M. A. Mullan J. T. (1981). Process the stress. Journal of Health and Social Behavior, 22(4), 337–356. 7320473

[bibr60-15579883211035241] Peter G. Bell M. M. Jarnagin S. Bauer D. (2009). Coming back across the fence: Masculinity and the transition to sustainable agriculture*. Rural Sociology, 65(2), 215–233. 10.1111/j.1549-0831.2000.tb00026.x

[bibr61-15579883211035241] Price L. Evans N. (2009). From stress to distress: Conceptualizing the British family farming patriarchal way of life. Journal of Rural Studies, 25(1), 1–11. 10.1016/j.jrurstud.2008.03.008

[bibr62-15579883211035241] Richardson N. (2010). “The ‘buck’ stops with me” - reconciling men’s lay conceptualisations of responsibility for health with men’s health policy. Health Sociology Review, 19(4), 419–436. 10.5172/hesr.2010.19.4.419

[bibr63-15579883211035241] Richardson N. Carroll P. C. (2009). Getting men’s health onto a policy agenda - charting the development of a National Men’s Health Policy in Ireland. Journal of Men’s Health, 6(2), 105–113. 10.1016/j.jomh.2009.03.004

[bibr64-15579883211035241] Rogers E. M. (1983). Diffusion of innovations. An integrated approach to communication theory and research (3rd ed.). Routledge. 10.4324/9780203710753-35

[bibr65-15579883211035241] Roy P. Hočevar D. K. (2019). Listening to a silent crisis: Men’s suicide in rural and farming communities in slovenia. Revija Za Socijalnu Politiku, 26(2), 241–254. 10.3935/rsp.v26i2.1593

[bibr66-15579883211035241] Roy P. Tremblay G. Oliffe J. L. Jbilou J. Robertson S. (2013). Male farmers with mental health disorders: A scoping review. Australian Journal of Rural Health, 21(1), 3–7. 10.1111/ajr.12008 23384130

[bibr67-15579883211035241] Roy P. Tremblay G. Robertson S. (2014). Help-seeking among male farmers: Connecting masculinities and mental health. Sociologia Ruralis, 54(4), 460–476. 10.1111/soru.12045

[bibr68-15579883211035241] Roy P. Tremblay G. Robertson S. Houle J. (2017). “Do it all by myself”: A salutogenic approach of masculine health practice among farming men coping with stress. American Journal of Men’s Health, 11(5), 1536–1546. 10.1177/1557988315619677 PMC5675206 26634855

[bibr69-15579883211035241] Shortall S. (2014). Farming, identity and well-being: Managing changing gender roles within Western European farm families. Anthropological Notebooks, 20(3), 67–81.

[bibr70-15579883211035241] Shortall S. Sutherland L.-A. McKee A. Hopkins J. (2017). Women in Farming and the Agriculture Sector. Rural and Environment Science and Analytical Services Division (RESAS), Scotland.

[bibr71-15579883211035241] Silverman D. (2000). Doing qualitative research Chapter 1. Doing qualitative research: A practical handbook. Sage, 1–12.

[bibr72-15579883211035241] Smyth B. Evans D. S. Kelly A. Cullen L. O’Donovan D. (2013). The farming population in Ireland: Mortality trends during the “celtic tiger” years. European Journal of Public Health, 23(1), 50–55. 10.1093/eurpub/cks017 22436692

[bibr73-15579883211035241] Storey A. Lane A. Cunningham C. McNamara J. (2014, March). An evaluation of the health behaviours of farmers in the South-East of Ireland [Conference session]. Agricultural Research Forum, Tullamore, IE, Europe. 10.13140/2.1.2095.9048

[bibr74-15579883211035241] Sunderman A. Johannes E. (2019). Investigation and response for farming-related stress among male farmers and their families. [Master of Science thesis]. Kansas State University Manhattan. https://krex.k-state.edu/dspace/handle/2097/40233

[bibr75-15579883211035241] Sutherland L. A. Burton R. (2011). Good farmers, good neighbours? The role of cultural capital in social capital development in a Scottish farming community. Sociologia Ruralis, 51(3), 238–255. 10.1111/j.1467-9523.2011.00536.x

[bibr76-15579883211035241] Sutherland L. A. Darnhofer I. (2012). Of organic farmers and “good farmers”: Changing habitus in rural England. Journal of Rural Studies, 28(3), 232–240. 10.1016/j.jrurstud.2012.03.003

[bibr77-15579883211035241] Tolley E. Robinson E. Priscillia U. (2016). Qualitative Methods in Public Health. Wiley.

[bibr78-15579883211035241] van Doorn D. Richardson N. Osborne A. (2017). Farmers have hearts: The prevalence of risk factors for cardiovascular disease among a subgroup of Irish livestock farmers. Journal of Agromedicine, 22(3), 264–274. 10.1080/1059924X.2017.1318728 28406370

[bibr79-15579883211035241] Verdonk P. Seesing H. De Rijk A. (2010). Doing masculinity, not doing health? A qualitative study among dutch male employees about health beliefs and workplace physical activity. BMC Public Health, 10(1), 712. 10.1186/1471-2458-10-712 21092090 PMC2998494

[bibr80-15579883211035241] Walker J. F. (2012). Mental health in the rural Sector: A review. http://www.mentalhealth.org.nz/assets/ResourceFinder/Mental-health-in-the-rural-sector-a-review-2012.pdf

[bibr81-15579883211035241] Ward N. (1993). The agricultural treadmill and the rural environment in the post-productivist era. Sociologia Ruralis, 33(3–4), 348–364. 10.1111/j.1467-9523.1993.tb00969.x

[bibr82-15579883211035241] Watson J. (2000). Male bodies: Health, culture and identity. In Open University Press. 10.1177/135910530100600610

[bibr83-15579883211035241] Wenger L. M. (2011). Beyond ballistics : Expanding our conceptualization of men’s health-related help seeking. American Journal of Men’s Health, 5(6), 488–499. 10.1177/1557988311409022 21659353

[bibr84-15579883211035241] World Health Organization. (2012). Public health action for the prevention of suicide: A framework. In World Health Organisation. https://doi.org/9789241503570

[bibr85-15579883211035241] World Health Organization. (2014). Preventing suicide: A global imperative. World Health Organization. https://apps.who.int/iris/handle/10665/131056

